# Biliverdin Reductase A (BVRA) Knockout in Adipocytes Induces Hypertrophy and Reduces Mitochondria in White Fat of Obese Mice

**DOI:** 10.3390/biom10030387

**Published:** 2020-03-02

**Authors:** David E. Stec, Darren M. Gordon, Andrea L. Nestor-Kalinoski, Matthew C. Donald, Zachary L. Mitchell, Justin F. Creeden, Terry D. Hinds

**Affiliations:** 1 Center for Excellence in Cardiovascular-Renal Research, Department of Physiology & Biophysics, University of Mississippi Medical Center, 2500 North State St, Jackson, MS 392161, USA; mcdonald@umc.edu (M.C.D.); zmitchell@umc.edu (Z.L.M.); 2 Center for Diabetes and Endocrine Research (CeDER), Department of Neurosciences, University of Toledo College of Medicine and Life Sciences, Toledo, OH 43614, USA; Darren.Gordon@rockets.utoledo.edu (D.M.G.); Justin.Creeden@rockets.utoledo.edu (J.F.C.); 3 Advanced Microscopy & Imaging Center, Department of Surgery, University of Toledo College of Medicine and Life Sciences, Toledo, OH 43614, USA; Andrea.Kalinoski@utoledo.edu

**Keywords:** obesity, WAT, BAT, adipose, brown fat, beige fat, bilirubin

## Abstract

Biliverdin reductase (BVR) is an enzymatic and signaling protein that has multifaceted roles in physiological systems. Despite the wealth of knowledge about BVR, no data exist regarding its actions in adipocytes. Here, we generated an adipose-specific deletion of biliverdin reductase-A (BVRA) (*Blvra*^FatKO^) in mice to determine the function of BVRA in adipocytes and how it may impact adipose tissue expansion. The *Blvra*^FatKO^ and littermate control (*Blvra*^Flox^) mice were placed on a high-fat diet (HFD) for 12 weeks. Body weights were measured weekly and body composition, fasting blood glucose and insulin levels were quantitated at the end of the 12 weeks. The data showed that the percent body fat and body weights did not differ between the groups; however, *Blvra*^FatKO^ mice had significantly higher visceral fat as compared to the *Blvra*^Flox^. The loss of adipocyte BVRA decreased the mitochondrial number in white adipose tissue (WAT), and increased inflammation and adipocyte size, but this was not observed in brown adipose tissue (BAT). There were genes significantly reduced in WAT that induce the browning effect such as *Ppara* and *Adrb3*, indicating that BVRA improves mitochondria function and beige-type white adipocytes. The *Blvra*^FatKO^ mice also had significantly higher fasting blood glucose levels and no changes in plasma insulin levels, which is indicative of decreased insulin signaling in WAT, as evidenced by reduced levels of phosphorylated AKT (pAKT) and Glut4 mRNA. These results demonstrate the essential role of BVRA in WAT in insulin signaling and adipocyte hypertrophy.

## 1. Introduction

Current estimates put the world’s obese population at one-third; however, that figure may be an underestimate of the global obesity epidemic [1,2,3]. Obesity is a co-morbidity for numerous pathological conditions, including cardiovascular disease, some cancers, increased traumatic injury, and inflammation [4,5,6]. To combat this epidemic, a better understanding of the factors that promote and protect against the harmful effects of obesity are needed.

Obesity increases insulin resistance and adipocyte size and reduces mitochondria number [7,8,9]. Adipocyte hypertrophy in obesity changes the profile of hormones and cytokines released, so-called “adipokines”. Excess fat in obesity is primarily stored in the viscera. The visceral fat releases more harmful adipokines than subcutaneous fat, resulting in worsened insulin resistance and inflammation, promoting complications such as type II diabetes and non-alcoholic fatty liver disease (NAFLD) [10,11]. Recent investigations have shown that biliverdin reductase-A (BVRA) can bind to the insulin receptor and increase sensitivity for glucose uptake [12,13], implicating it may be a potential therapeutic for reversing glucose intolerance.

Biliverdin reductase (BVR) is the enzyme responsible for the reduction of biliverdin to bilirubin [14,15,16,17]. It consists of two isoforms BVRA and biliverdin reductase-B (BVRB), which are expressed at different times in development [17]. BVRB is the main isoform expressed embryonically, and BVRA is expressed after fetal development [17]. In adults, the BVRA isozyme is essential, as it reduces biliverdin IXα to bilirubin IXα, which at this age is the only version present [17]. Recently, BVRA was shown to be significantly lower in peripheral blood mononuclear cells (PBMC) from obese humans compared to matched lean controls [18]. Others have described that BVRA mediates the direction of macrophage chemotaxis and polarization [19,20]. Our studies have shown that the BVRA isoform is essential for protection from hepatic steatosis and insulin resistance through its positive regulation of nuclear receptor peroxisome proliferator-activated receptor-α (PPARα) [21]. PPARɑα regulates fat accumulation by inducing fat-burning genes in the liver [22] and improving mitochondrial function in white adipocytes by stimulating ‘browning’ or ‘beiging,’ which reduces body weight [23]. Drugs that target mitochondrial respiration through upregulation of mitochondrial uncoupling proteins (UCPs) can promote the “browning” of white adipose tissue (WAT), which decreases the storage of fat and increases fat burning within the WAT [24,25]. The role of BVRA in control of adipocyte mitochondrial function or beiging is unknown. Here, we report the characterization of BVRA in adipocytes.

## 2. Materials and Methods

### 2.1. Animals

**Figure 1:**
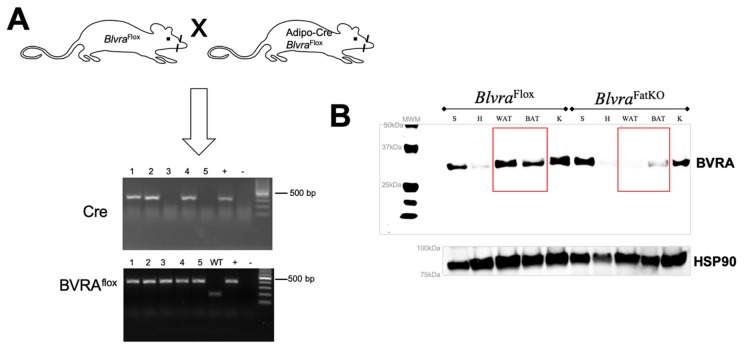
Adipose-specific knockout of biliverdin reductase-A (BVRA) in mice. (**A**) Homozygous floxed BVRA (*Blvra*^Flox^) mice were crossed to mice expressing the Cre recombinase specifically in adipose under the control of the adiponectin promoter to create adipose-specific BVRA knockout mice (*Blvra*^FatKO^). (**B**) Western blot for biliverdin reductase-A (BVRA) and heat shock protein 90 (HSP90) as a loading control in various tissues from *Blvra*^Flox^ and *Blvra*^FatKO^ mice. WAT, white adipose tissue. BAT, brown adipose tissue.

The experimental procedures and protocols of this study conformed to the National Institutes of Health Guide for the Care and Use of Laboratory Animals and were approved by the Institutional Animal Care and Use Committee of the University of Mississippi Medical Center. BVRA conditional knockout mice were generated from gene-targeted embryonic stem cells as previously described [21]. For adipose-specific knockout, homozygous floxed BVRA (*Blvra*^Flox^) mice were crossed to mice expressing the Cre recombinase specifically in adipose under the control of the adiponectin promoter (stock # 028020, Jackson Labs, Bar Harbor, ME, USA) to create adipose-specific BVRA knockout mice (*Blvra*^FatKO^). Breeding pairs consisted of a homozygous *Blvra*^Flox^ mouse crossed with a homozygous *Blvra*^Flox^ mouse heterozygous for the Cre allele. This breeding strategy generated litters containing both *Blvra*^Flox^ and *Blvra*^FatKO^ littermates (Figure 1). All mice were on a C57BL/6J genetic background. Studies were performed on separate cohorts of 6-week old male housed under standard conditions with full access to standard mouse chow and water and maintained at an ambient temperature of 24 °C. Mice were house between 2–5 mice per cage. After this time mice were switched to a 60% high-fat diet (diet # D12492, Research Diets, Inc., New Brunswick, NJ, USA) for 12 weeks and allowed access to water. Mice were euthanized and tissues and plasma samples collected after an 8 h fast. White adipose tissue (WAT) was collected from samples of epididymal fat and brown adipose tissue (BAT) was collected from the area between the scapula. Tissues were immediately frozen in liquid nitrogen and stored at −80 °C.

### 2.2. Body Composition

Body composition (fat mass, free water, and total water) was measured at 4-week intervals throughout the 12-week study using magnetic resonance imaging (EchoMRI-900TM, Echo Medical System, Houston, TX, USA) as previously described [26,27]. Body composition analysis was measured in conscious mice exposed to a low-intensity electromagnetic field. Mice were placed in a thin-walled plastic cylinder with a cylindrical plastic insert that functioned to limit the movement of the mice while they were in the EchoMRI instrument.

### 2.3. Fasting Glucose and Insulin

Fasting blood glucose and insulin were obtained from plasma samples collected following an 8 h fast. Samples were collected under light isoflurane anesthesia via the orbital sinus. The fasting glucose levels were measured using an Accu-Chek Advantage glucometer (Roche, Mannheim, Germany) and fasting insulin levels were measured by ELISA (Ultrasensitive Mouse Insulin Kit, Crystal Chem, Elk Grove Village, IL, USA) as previously described [22,26,28,29,30,31].

### 2.4. Quantitative Real-Time PCR Analysis

WAT and BAT specimens from *Blvra*^FatKO^ and *Blvra*^Flox^ mice were prepared for RNA extrations by placement of tissues in QIAzol Lysis Reagent (Qiagen, Germantown, MD, USA) using a Qiagen TissueLyser LT (Qiagen, Germantown, MD, USA) with a setting of 50 oscillations per second for 3 min. Samples were then passed through the total RNA extraction column and procedure followed as described for the miRNeasy Mini Kit (Qiagen, Germantown, MD, USA). Total RNA was read on a NanoDrop 2000 spectrophotometer (Thermo Fisher Scientific, Wilmington, DE, USA) and cDNA was synthesized using High Capacity cDNA Reverse Transcription Kit (Applied Biosystems, Wilmington, DE, USA). PCR amplification of the cDNA was performed by quantitative real time PCR using TrueAmp SYBR Green qPCR SuperMix (Advance Bioscience, Edwards, CO, USA). The thermocycling protocol consisted of 3 min at 95 °C, 48 cycles of 15 sec at 95 °C, 30 sec at 60 °C, and based on primer size 0 to 30 sec at 72 °C and finished with a melting curve ranging from 60–95 °C to allow distinction of specific products. Normalization was performed in separate reactions with primers to 36B4, which is an endogenous housekeeping gene previously described in [32,33].

### 2.5. Gel Electrophoresis and Western Blotting

Tissue lysates were prepared from WAT specimens from *Blvra*^FatKO^ and *Blvra*^Flox^ mice were suspended in CelLytic MT Cell Lysis Reagent Buffer for mammalian tissues (Millipore Sigma, St. Louis, MO, USA, Cat No: C3228) that contained protease inhibitor cocktail (Millipore Sigma, St. Louis, MO, USA, Cat No: P2714-1BTL) and Halt phosphatase inhibitor (Thermo Fisher Scientific, Wilmington, DE, USA, product # 1862495) using a Qiagen TissueLyser LT (Qiagen, Germantown, MD, USA) with a setting of 50 oscillations per second for 3 min. As previously described [31,34,35,36,37,38,39], lysates were centrifuged at 100,000× *g* for 7 min and the supernatant removed and measured for protein concentration by BCA Protein Assay Kit (Thermo Fisher Scientific, Wilmington, DE, USA). Protein specimens were resolved by SDS polyacrylamide gel electrophoresis and electrophoretically transferred to Immobilon FL membranes. Membranes were blocked at room temperature for 1 h in Odyssey Blocking buffer (LI-COR Biosciences, Lincoln, NE, USA) or TBS (TBS; 10 mM Tris HCl (pH 7.4) and 150 mM NaCl) containing 5% BSA or milk. Subsequently, the membrane was incubated overnight at 4 °C with antibodies for BVRA (Enzo, Farmingdale, NY, USA, ADI-OSA-450-E), phospho-Akt (pSer473) (Cell Signaling Technology, Danvers, MA, USA, 4060s), AKT1/2 (Santa Cruz Biotechnology, Dallas, TX, USA, sc-1619) or HSP90 antibodies (Santa Cruz Biotechnology, Dallas, TX, USA, sc-13119). After three washes in TBST (TBS plus 0.1% Tween 20), the membrane was incubated with an infrared anti rabbit (IRDye 800, green) or anti mouse (IRDye 680, red) secondary antibody labeled with IRDye infrared dye (LI COR Biosciences, Lincoln, NE, USA) (1:15,000 dilution in TBS) for 2 h at 4 °C. Following an additional 3 washes in TBST, immunoreactivity was visualized and quantified by infrared scanning in the Odyssey system (LI COR Biosciences, Lincoln, NE, USA).

### 2.6. Measurement of Mitochondrial Density and Lipid Droplet Sizes

To determine mitochondrial numbers, we used cryopreserved intact native adipose tissue as described by Fuller et al. [40]. WAT and BAT samples from the *Blvra*^FatKO^ and *Blvra*^Flox^ mice were thawed at room temperature in prewarmed 37 °C PBS and washed three times for preparation of imaging as we have previously described [41]. Samples were incubated with 100nM Mitotracker^®^ Green FM (M7514, Molecular Probes, Eugene, OR, USA) for 15 min at room temperature. WAT Mitotracker staining was previously described in [42]. The samples were washed once with PBS, then incubated for 5 min with 1 μM of Draq-5 (Cell Signaling Technology, Danvers, MA, USA). Adipose tissue was washed one final time with PBS before imaging using a Leica TSC SP5 laser scanning confocal microscope in 1 µm steps. Samples were imaged using the 488 and 633 laser lines for excitation with peak emission collection at 514 and 647 respectively.

Brightfield images of the lipid droplet sizes were measured as previously described [43]. Tissue sample diameters (d) were quantitated based on the measurement of the lipid droplet’s widest point in LAS AF software (Leica Microsystems, Buffalo Grove, IL, USA). The diameter was used to extrapolate the lipid area for the adipocytes using the formula: πr^2^ where r = ½ *d*.

### 2.7. Statistics

Data are expressed as mean + SEM. Data were analyzed using analysis or variance and a Tukey’s post-test was utilized to compare group means utilizing Prism 7 (GraphPad Software, San Diego, CA, USA). *p* values of 0.05 or smaller were considered statistically significant.

## 3. Results

### 3.1. Selective Deletion of BVRA in Adipose Tissue in Mice

To generate an adipose-specific BVRA knockout (*Blvra*^FatKO^), homozygous floxed BVRA (*Blvra*^Flox^) mice were crossed with mice expressing Cre recombinase under the control of the adiponectin promoter (Figure 1A). Western blot analysis of the levels of BVRA expressed in various tissues, including spleen, heart, white adipose (WAT), brown adipose (BAT), and kidney showed that *Blvra*^FatKO^ mice exhibited near-complete loss of BVRA in the WAT (reduced 97.7%) and BAT (reduced 72.7%) as compared to the *Blvra*^Flox^ mice (Figure 1B). BVRA levels in other tissues such as the spleen, heart, and kidney were not different between the two groups of mice.

### 3.2. Blvra^FatKO^ Mice Exhibit Greater Levels of Visceral Fat as Compared to Littermate Controls

**Figure 2:**
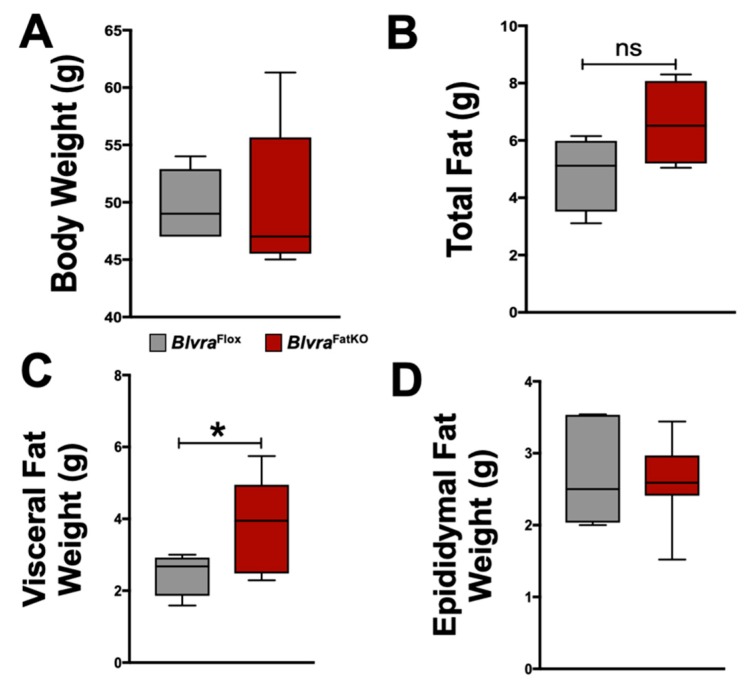
Bodyweight and fat distribution in *Blvra*^Flox^ and *Blvra*^FatKO^ mice fed a high-fat diet. (**A**) Body weights at the end of the 12-week HFD; (**B**) Total fat at the end of the 12-week HFD; (**C**) Visceral Fat at the end of the 12-week HFD; (**D**) Epididymal fat at the end of the 12-week HFD. *, *p* < 0.05 (vs. *Blvra*^Flox^). *n* = 5/group *Blvra*^Flox^; *n* = 7/group *Blvra*^FatKO^.

At the end of the 12-week high-fat diet (HFD) feeding period, body weights (Figure 2A), body fat and lean percentages (Table 1), and non-adipocyte tissue weights (Table 1) were similar between the *Blvra*^Flox^ and *Blvra*^FatKO^ mice. Despite these similarities, *Blvra*^FatKO^ mice exhibited a trend towards increased total fat measured at the time of euthanasia and a significant increase in visceral fat with no difference in epididymal fat between the *Blvra*^Flox^ and *Blvra*^FatKO^ mice (Figure 2B–D). Total body fat, as measured by non-invasive echo MRI at the end of the 12-week HFD, was also not different between *Blvra*^Flox^ and *Blvra*^FatKO^ mice.

### 3.3. Loss of Adipose BVRA Decreases Insulin Signaling and Elevates Inflammatory Pathways in White Adipocytes

**Figure 3:**
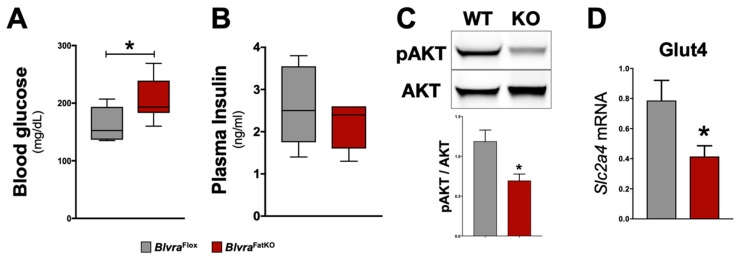
Insulin signaling pathway in *Blvra*^Flox^ and *Blvra*^FatKO^ mice fed a high-fat diet. (**A**) Fasting blood glucose levels at the end of the 12-week HFD; (**B**) Fasting blood insulin levels at the end of the 12-week HFD; (**C**) Representative Western blot of phospho-Akt (pSer473) and total AKT protein levels in the adipose tissue; (**D**) *Slc2a4* mRNA (also known as Glut4) expression. *, *p* < 0.05 (vs. *Blvra*^Flox^). *n* = 5/group *Blvra*^Flox^; *n* = 7/group *Blvra*^FatKO^.

**Figure 4:**
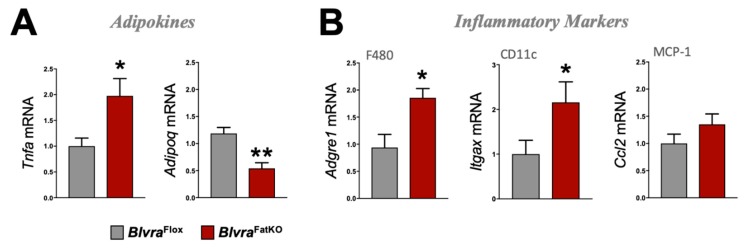
Adipokine and inflammatory marker expression in WAT from *Blvra*^Flox^ and *Blvra*^FatKO^ mice fed a high-fat diet. (**A**) *Tnfa* and *Adipoq*, and (**B**) *Adgre1*, *Itgax*, and *Ccl2* mRNA expression in WAT. *, *p* < 0.05 (vs. *Blvra*^Flox^). *n* = 4/group *Blvra*^Flox^; *n* = 5/group *Blvra*^FatKO^.

The deletion of BVRA in the adipose tissue increased fasting blood glucose levels measured at the end of the study in *Blvra*^FatKO^ as compared to *Blvra*^Flox^ mice (Figure 3A). The increase in fasting hyperglycemia observed in the *Blvra*^FatKO^ mice did not correlate with any changes in fasting insulin levels between the groups (Figure 3B). This data demonstrates a decrease in insulin signaling in the *Blvra*^FatKO^ mice. The phosphoinositide 3-kinase (PI3K)/AKT signaling pathway is required for normal metabolism, and its imbalance leads to the development of obesity and type-2 diabetes mellitus. The PI3K/AKT pathway is critical for insulin signaling; hence, any defect in AKT/PKB pathway along with the downstream molecules may lead to insulin resistance. We measured the levels of phosphorylated AKT (pAKT) and total AKT as well as Glut4 mRNA levels from WAT of *Blvra*^Flox^ and *Blvra*^FatKO^ mice. The *Blvra*^FatKO^ mice exhibit significantly reduced levels of pAKT and Glut4 mRNA as compared to *Blvra*^Flox^ mice (Figure 3C,D). The loss of BVRA significantly increased proinflammatory TNFɑ (*Tnfa*) and reduced anti-inflammatory adiponectin (*Adipoq*) adipokines (Figure 4A). This was pararlleled with significantly higher inflammatory markers F480 (*Adgre1*) and CD11c (*Itgax*) (Figure 4B).

### 3.4. Adipose Knockout of BVRA Reduces Mitochondrial Number and Beige Fat Markers in WAT But Not BAT

**Figure 5:**
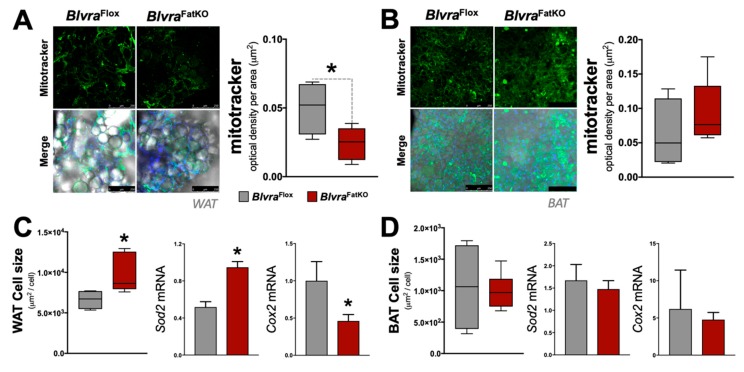
Mitochondrial levels and adipocyte size in white adipose tissue (WAT) and brown adipose tissue (BAT) from *Blvra*^Flox^ and *Blvra*^FatKO^ mice fed a high-fat diet. (**A**) Mitochondrial density (µm^2^) in WAT; (**B**) Mitochondrial density (µm^2^) in BAT. *Scale bars*, 250 µM; (**C**) Adipocyte size (µm^2^/cell) in WAT and mRNA expression of *Sod2* and *Cox2*; (**D**) Adipocyte size (µm^2^/cell) in BAT and mRNA expression of *Sod2* and *Cox2*. *, *p* < 0.05 (vs. *Blvra*^Flox^). *n* = 4/group *Blvra*^Flox^; *n* = 5/group *Blvra*^FatKO^.

**Figure 6:**
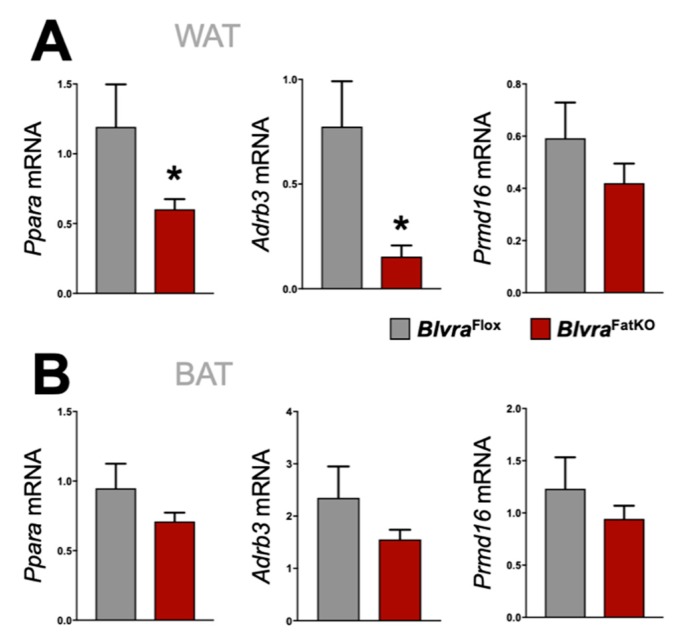
Beiging gene marker expression in WAT and BAT from *Blvra*^Flox^ and *Blvra*^FatKO^ mice fed a high-fat diet. *Ppara*, *Adrb3*, and *Prmd16* mRNA expression in WAT (**A**) and BAT (**B**). *, *p* < 0.05 (vs. *Blvra*^Flox^). *n* = 4/group *Blvra*^Flox^; *n* = 5/group *Blvra*^FatKO^.

Mitochondria play an important role in the physiology of adipocytes. To determine the effect of the loss of adipose BVRA on mitochondria, we utilized the Mitotracker mitochondrial staining technique to measure the number [42] in the WAT and BAT in *Blvra*^Flox^ and *Blvra*^FatKO^ mice. The deletion of BVRA from the adipose tissues resulted in a significant decrease in mitochondrial number in WAT of *Blvra*^FatKO^ as compared to *Blvra*^Flox^ mice (Figure 5A). However, the loss of BVRA did not affect the amount of mitochondria in BAT (Figure 5B). The adipocyte size was significantly larger in WAT of the *Blvra*^FatKO^ as compared to *Blvra*^Flox^ mice (Figure 5C), but this was not observed in BAT (Figure 5D). Also, *Sod2* was significantly increased in WAT, indicating oxidative stress levels were high, which is known to be heightened by inflammatory stimuli [44]. Similar to the mitotracker staining, *Cox2,* a gene known to control mitochondria in WAT [45], was significantly reduced. These indicate that the loss of BVRA in WAT causes whitening and increased WAT size, reducing mitochondria levels. Further indicators of this are reduced beiging markers *Ppara* and *Adrb3* in WAT of the *Blvra*^FatKO^ compared to the *Blvra*^Flox^ (Figure 6A). However, *Prmd16*, another beige fat marker, was unchanged between the groups. There was no significant difference for *Ppara*, *Adrb3*, or *Prmd16* in BAT between the groups (Figure 6B).

## 4. Discussion

Our results demonstrate that while the loss of adipocyte BVRA does not affect the total amount of fat gained in response to a HFD, it does play a role in the distribution of fat, resulting in considerably more visceral fat. Thus, increased expansion of visceral fat in the *Blvra*^FatKO^ mice may contribute to the higher glucose and lower pAKT-insulin signaling observed in these mice fed a HFD. Visceral fat releases greater amounts of damaging adipokines (TNFα), which can result in insulin resistance and inflammation [10,11,46,47]. In humans, lower BVRA levels are found in obesity and contribute to the metabolic syndrome and visceral adipose tissue inflammation [18]. Not surprisingly, BVRA has anti-inflammatory actions via inhibition of toll-like receptor 4 (TLR4) and NF-κB [48,49,50,51,52]. Weigel et al. showed that BVRA mediates biliverdin-induced anti-inflammatory effects via phosphatidylinositol 3-kinase and AKT (PI3K/AKT). Here, we found a similar finding that the adipocyte-loss of BVRA caused WAT inflammation and reduced pAKT, as well as lowered mitochondria number and increased adipocyte size.

The PI3K/AKT signaling pathway regulates insulin action in many tissues, including adipose and skeletal muscle, where it promotes glucose transport, glycogen synthesis, and protein synthesis [53,54]. Tonks et al. demonstrated that insulin-stimulated AKT phosphorylation at Thr309 and Ser474 highly correlated with whole-body insulin sensitivity in overweight/obese type 2 diabetic patients before and during a hyperinsulinaemic-euglycaemic clamp [55]. Given the importance of this pathway to the actions of insulin, Zhang et al. proposed activation of AKT as a novel strategy to treat insulin resistance [56]. One of the primary regulators of AKT activity and insulin signaling is BVRA [18,57]. Studies by Miralem et al. elegantly demonstrated that BVRA modulates AKT activity by aiding the formation of a complex with phosphatidylinositol-dependent kinase 1 (PDK1) [12]. The interaction of BVRA with AKT increases activity and phosphorylation (pAKT), improving insulin sensitivity [12,21]. Specific peptide sequences within the BVRA protein itself can impact insulin sensitivity through activation or inhibition of the insulin receptor kinase (IRK) domain [58]. The delivery of nanoparticles corresponding to the C-terminal KYCCSRK peptide sequence of human BVR improves glucose clearance in obese *ob/ob* mice by activation of the pAKT pathway [59]. Thus, it is clear that BVRA can impact insulin signaling through its interactions with AKT. Others have shown that the loss of BVRA in the brain causes insulin-resistance that occurs in Alzheimer’s disease [60,61,62]. However, the loss of BVRA in obesity can result in hyperactivation of insulin signaling [18]. These conflicting results highlight the lack of consensus in the field. Our study is the first to demonstrate that the adipose-specific loss of BVRA decreases the PI3K/AKT pathway. The deletion of WAT BVRA resulted in the attenuation of pAKT, which may contribute to the reduced insulin signaling and higher blood glucose and inflammation exhibited in the *Blvra*^FatKO^ mice. Further studies in both animal models and humans are needed to understand the complex relationship between BVRA and AKT fully. Limitations of the current study are the lack of comparison to mice fed a normal fat diet, and that food intake was not measured. Thus, the role of adipose BVRA on the adaptive changes that occur in response to high-fat feeding could not be determined in the present study.

Mitochondria play a vital role in the maintenance of adipocyte function. Strategies to increase the “browning” or “beiging” of adipose tissue, are now being considered as potential treatments for obesity [63,64,65]. In the present study, the loss of BVRA from WAT resulted in a reduction of mitochondria number. This result is consistent with previous studies in which the deletion of BVRA in both cultured renal proximal tubule cells, as well as cultured hepatocytes, resulted in the decrease in mitochondrial number, mitochondrial membrane potential, oxygen consumption, and extracellular acidification rate [66,67]. Furthermore, the loss of BVRA was shown to decrease the expression of several mitochondrial complex subunit genes as well as mitochondrial dynamin-like GTPase (Opa1), which codes for a protein of the inner mitochondrial membrane regulating mitochondrial stability and energy output [67]. The results of these studies highlight the important role of BVRA in the maintenance of mitochondrial function, which is surprising given the fact that BVRA does not localize to mitochondria [17]. If BVRA is not found in mitochondria, then why does the loss of BVRA have such a great impact on mitochondrial function? There are several potential mechanisms by which this may occur, including increased reactive oxygen species production in response to BVRA deletion [19,66,68,69]. In the present study, decreases in the metabolite of BVRA, bilirubin, may be responsible for this observed effect on mitochondria number in *Blvra*^FatKO^.

We have previously demonstrated that hepatocyte-specific loss of BVRA results in exacerbation of hepatic steatosis and insulin resistance in response to a chronic HFD through alterations in PPARɑ [21]. Activation of heme oxygenase-1 (HO-1) has beneficial effects to prevent fatty liver disease [15,31,70,71], which is most likely mediated by BVRA production of bilirubin [15,21,28,72]. Others have shown that heme-derived metabolic signals dictate immune responses [21]. Bilirubin has strong antioxidant properties, and we have shown that it also signals through the nuclear hormone receptor PPARɑ to up-regulate genes associated with increased fatty acid oxidation and glucose sensitization [72,73]. Bilividen is rapidly converted to bilirubin by BVRA [74], and biliverdin treatment in diabetic [75] or obese [76] mice improves metabolic dysfunction. However, biliverdin treatment in human HepG2 hepatocytes with PPARɑ knockdown attenuates transcriptome responses [73]. PPARɑ associates with peroxisome proliferator-activated receptor gamma coactivator *1*-alpha (*PGC*-1ɑ) to increase mitochondrial biogenesis [77,78]. Here, we found that BVRA in WAT, like liver, also regulates PPARɑ expression (*Ppara* mRNA) and known beiging target gene *Adrb3*. It is interesting that while similar mechanisms by which bilirubin regulates mitochondrial function are present in BAT, the deletion of BVRA did not affect mitochondrial numbers in BAT in the *Blvra*^FatKO^ mice. The exact mechanism by which brown fat escapes this BVRA influence remains unknown. BAT may exhibit redundant pathways (i.e., greater sympathetic input) that are not present in WAT, which serves to preserve mitochondrial numbers and function, or the loss of BVRA in BAT may not have had an impact on inflammatory stimuli such as that observed in WAT. The mechanism(s) that protect mitochondrial numbers in BAT following the deletion of BVRA requires further study.

## 5. Conclusions

In summary, adipocyte-specific deletion of BVRA caused increased expansion of visceral fat adipocyte size and inflammation, and reduced insulin signaling and mitochondria number. At the same time, this deletion did not have a direct impact on weight gain in response to chronic HFD feeding. The loss of BVRA in WAT decreased pAKT levels, which may contribute to insulin resistance in humans, but this topic requires further study. Our results demonstrate the notable regulatory role of BVRA in WAT and strongly suggest that BVRA may contribute to beiging and increased mitochondrial functionality, and inhibit inflammation. Increasing BVRA in WAT may provide a novel therapeutic target for the improvement of obesity-associated insulin resistance, inflammation, and adipocyte hypertrophy.

## Figures and Tables

**Table 1:** Fat and lean mass percentage and tissue weights in *Blvra*^Flox^ and *Blvra*^FatKO^.

Parameter	*Blvra*^Flox^ (n = 5)	*Blvra*^FatKO^ (n = 7)	*p* Value
Fat Mass (%)	46.5 ± 0.7	44.7 ± 1.5	0.2999
Lean Mass (%)	51.3 ± 0.8	53.3 ± 1.4	0.2556
Body Length (cm)	10 ± 0.15	10.2 ± 0.15	0.313
Tibia Length (cm)	2.2 ± 0.03	2.3 ± 0.02	0.109
Heart Weight (mg)	145.1 ± 14.2	139.6 ± 7.4	0.6573
Heart Weight/Body Weight (mg/g)	3.3 ± 0.3	2.9 ± 0.15	0.2687
Heart Weight/Body Length (mg/cm)	14.5 ± 1.3	13.6 ± 0.6	0.5
Heart Weight/Tibia Length (mg/cm)	62.9 ± 4.3	59.4 ± 3.4	0.5126
Kidney Weight (mg)	328 ± 15	328 ± 12	0.9945
Kidney Weight/Body Weight (mg/g)	7.4 ± 0.5	6.9 ± 0.3	0.3070
Kidney Weight/Body Length (mg/cm)	32.8 ± 1.4	31.7 ± 1.1	0.6650
Kidney Weight/Tibia Length (mg/cm)	145.8 ± 6	140.1 ± 5	0.4227
Liver Weight (g)	1.8 ± 0.3	1.7 ± 0.2	0.7989
Liver Weight/Body Weight (mg/g)	0.03 ± 0.005	0.03 ± 0.001	0.4724
Liver Weight/Body Length (mg/cm)	0.17 ± 0.03	0.16 ± 0.01	0.6972
Liver Weight/Tibia Length (mg/cm)	0.71 ± 0.13	0.69 ± 0.03	0.8618
